# Newborn Screening for Severe Combined Immunodeficiency Using the Multiple of the Median Values of T-Cell Receptor Excision Circles

**DOI:** 10.3390/ijns7030043

**Published:** 2021-07-12

**Authors:** Michael F. Cogley, Amy E. Wiberley-Bradford, Sean T. Mochal, Sandra J. Dawe, Zachary D. Piro, Mei W. Baker

**Affiliations:** 1 Newborn Screening Laboratory, Wisconsin State Laboratory of Hygiene, University of Wisconsin School of Medicine and Public Health, Madison, WI 53726, USA; michael.cogley@slh.wisc.edu (M.F.C.); amy.wiberley-bradford@slh.wisc.edu (A.E.W.-B.); sean.mochal@slh.wisc.edu (S.T.M.); zachary.piro@slh.wisc.edu (Z.D.P.); 2 Office of Information Systems, Wisconsin State Laboratory of Hygiene, University of Wisconsin School of Medicine and Public Health, Madison, WI 53726, USA; sam.dawe@slh.wisc.edu; 3 Genetics and Metabolism Division, Department of Pediatrics, University of Wisconsin School of Medicine and Public Health, Madison, WI 53726, USA; 4 Center for Human Genomics and Precision Medicine, University of Wisconsin School of Medicine and Public Health, Madison, WI 53726, USA

**Keywords:** severe combined immunodeficiency, multiple of the median, newborn screening, T-cell receptor excision circles

## Abstract

All newborn screening programs screen for severe combined immunodeficiency by measurement of T-cell receptor excision circles (TRECs). Herein, we report our experience of reporting TREC assay results as multiple of the median (MoM) rather than using conventional copy numbers. This modification simplifies the assay by eliminating the need for standards with known TREC copy numbers. Furthermore, since MoM is a measure of how far an individual test result deviates from the median, it allows normalization of TREC assay data from different laboratories, so that individual test results can be compared regardless of the particular method, assay, or reagents used.

## 1. Introduction

Severe combined immunodeficiency (SCID) is a group of disorders caused by at least 14 single-gene variants, all of which cause defects in the development of normal naive T-cells. This leads to combined cellular and humoral immunodeficiency. Infants with SCID typically appear healthy at birth. Undiagnosed and untreated, infection-related death typically occurs by one to two years of age. Early diagnosis and treatment of SCID by hematopoietic stem cell transplantation or gene therapy are essential to prevent death and to establish a normal functional immune system [1]. Published data have demonstrated that the measurement of T-cell receptor excision circles (TRECs) by real-time qPCR can successfully identify infants with SCID [2]. TRECs are small-circle DNA molecules that are byproducts of T-cell maturation in the thymus, and their quantity reflects the number of T-cells recently emigrated from the thymus [3]. Since all infants with SCID have a profound decrease in T-lymphocytes regardless of what pathogenic variants are involved, the quantity of TRECs present in dried blood specimens collected one to two days post-delivery from SCID babies is very low when compared to healthy newborns [4]. In 2008, the Wisconsin Newborn Screening (NBS) program implemented a statewide NBS program for SCID [5]. In 2010, SCID screening was added to the Recommended Uniform Screening Panel [6]. By the end of 2018, all NBS programs in the United States had added SCID to their screening panels. Progress in population-based NBS for SCID has also been made in many other countries, including Saudi Arabia, Spain, France, Sweden, the Netherlands, Taiwan, Brazil, Japan, and Israel [7,8,9,10,11,12,13,14,15].

Our program’s NBS for SCID initially reported TREC results quantitatively (T-cell receptor excision circles per microliter of blood) based on serial dilutions of plasmids containing known TREC copy numbers [4]. Over time, we experienced increasing challenges in maintaining stable, consistent TREC concentrations in plasmids. Intrigued by a presentation at an Association of Public Health Laboratories NBS conference that introduced the concept of using multiple of the median (MoM) values for reporting TREC assay results [16], we started to explore the use of this metric. The MoM is defined as the ratio of the result of an individual measurement to the median result for measurements in the appropriate population; it has been widely used to report maternal serum alpha-fetoprotein results in prenatal screening for Down syndrome and neural tube defects [17,18]. Herein, we report our experience of using TREC MoM values in our SCID screening program.

## 2. Materials and Methods

A total of 157,172 Wisconsin newborns born between 1 September 2018 and 31 March 2021 underwent NBS for SCID. The demographic information of these newborns is summarized in Table 1.

### 2.1. Samples

The newborn specimens consisted of whole blood, collected by heel stick onto approved filter paper card 24–48 h after birth. The specimen cards were allowed to dry prior to transport. No newborn specimens were collected on site. Specimens were sent from the site of collection to the laboratory for testing.

### 2.2. DNA Isolation

DNA was isolated from routine NBS dried blood spot (DBS) specimens using Extracta DBS (Quantabio, Beverly, MA, USA). The 3.2 mm DBS punches in a 96-well plate were washed in 90 µL of Extracta DBS solution. They were spun in a centrifuge for 5 min at 2250× *g* and the supernatant was removed. To elute the DNA from DBS, 54 µL of Extracta DBS solution was added to each well and the plates were sealed and incubated for 25 min at 96 °C and then brought to 4 °C.

### 2.3. Real-Time PCR Assay

Our lab-developed multiplex real-time PCR assay measures TREC and survival motor neuron 1 (*SMN1*), with *RPP30* as a reference gene. This assay was designed for simultaneous screening for SCID and spinal muscular atrophy (SMA). The primer, probe (custom synthesized by Integrated DNA Technologies, Coralville, IA, USA), and blocker (custom synthesized by Qiagen, Germantown, MD, USA) sequences are listed in Table 2. Each 20 µL reaction mixture contained 1x Quanta Multiplex Toughmix (Quantabio), 300 nM TREC primers, 46.875 nM *SMN* primers, 25 nM *RPP30* primers, 150 nM TREC probe, 56.25 nM *SMN1* probe, 56.25 nM *SMN2* blocker, 75 nM *RPP30* probe, 0.57 mg/mL of bovine serum albumin (New England Biolabs, Ipswich, MA), and 6 µL of DNA extract. The following thermal profile was used for amplification: 5 min at 94 °C, followed by 40 cycles of 15 s at 94 °C, 33 s at 60 °C, and 40 s at 68 °C. The reactions were run on a QuantStudio^TM^ 5 Real-Time PCR system (Thermo Fisher Scientific, Waltham, MA, USA), and cycle threshold (Ct) values for all amplicons were displayed by the instrument software at a fixed fluorescent signal threshold in the exponential phase of amplification. These Ct values were used directly to calculate the MoM (Ct_individual’s value_ ÷ Ct_population median_). The SMA screening incorporated into this assay is not relevant to this manuscript.

### 2.4. MoM Cutoff Establishment

A set of 2244 de-identified residual DBS specimens from routine NBS was assayed to assess the distribution of the TREC and RPP30 values in the population. To evaluate the clinical validity of the TREC and RPP30 cutoffs, residual routine NBS specimens from 19 confirmed cases of SCID, 22q11.2 deletion syndrome, and other lymphopenias, as well as 75 residual proficiency testing specimens, were assayed.

## 3. Results

### 3.1. Results of Cutoff Establishment

The 2244 specimens analyzed for cutoff establishment were found to have a mean TREC Ct of 30.762 and a median TREC Ct of 30.705. The same samples had mean and median RPP30 Ct values of 24.28 and 24.243, respectively (Table 3). A MoM of 1.079, the 99th percentile of the tested samples, was set for the TREC cutoff. A MoM of 1.035, the 90th percentile of the tested samples, was set for the RPP30 cutoff.

When residual NBS specimens from confirmed lymphopenia cases and from proficiency testing specimens were analyzed, it was found that setting the cutoff for a positive screen at a MoM of 1.079 would identify every sample that had previously screened positive. It was also indicated that setting the RPP30 cutoff at a MoM of 1.035 would identify specimens with poor DNA quality or quantity that required recollection.

### 3.2. SCID Screening Algorithm and Its Results

**Figure 1:**
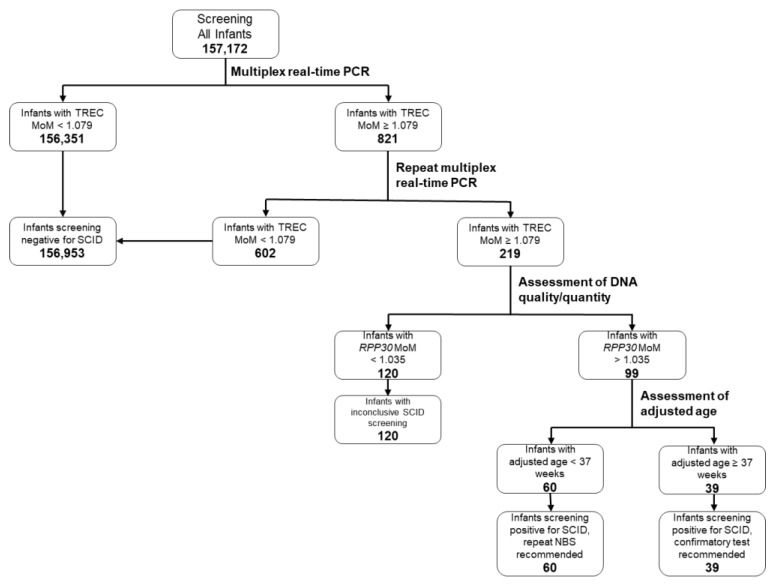
Wisconsin SCID newborn screening algorithm.

The Wisconsin SCID screening algorithm is summarized in Figure 1. Newborns with a TREC MoM <1.079 are deemed to screen negative for SCID. Newborns with a TREC MoM value ≥1.079 upon first analysis are re-tested in duplicate, with two new punches taken from the same specimen card. If both of these samples have a TREC MoM <1.079, the newborn is deemed to screen negative for SCID. If the repeat samples have a TREC MoM ≥1.079, DNA quality and quantity are assessed by analysis of the *RPP30* MoM. If the *RPP30* MoM exceeds 1.035, the screen is deemed inconclusive, and a repeat newborn screen is recommended. If the *RPP30* MoM is less than 1.035, the screen is deemed positive, and the recommended action is based on the newborn’s adjusted age (gestational age plus age at collection). For newborns with an adjusted age of <37 weeks, a repeat newborn screen is recommended. For newborns with an adjusted age of ≥37 weeks, confirmatory testing is recommended.

Of the 157,172 infants screened for SCID between September 2018 and March 2021, 156,953 had a TREC MoM value of <1.079 on either the initial assay or when subsequently re-tested in duplicate. This resulted in 99.86% of infants screening negative for SCID. Another 120 infants had inconclusive screens, with a TREC MoM ≥1.079 and a *RPP30* MoM >1.035, and thus repeat NBS was recommended for these infants. Finally, 99 infants screened positive for SCID. Of these, 60 had adjusted ages of <37 weeks and had repeat NBS recommended. The remaining 39 infants, whose adjusted ages were ≥37 weeks, were referred for confirmatory testing based on the results of their initial NBS for SCID (Figure 1).

Of the 120 infants with an inconclusive first SCID screening, 105 screened negative for SCID when a second NBS specimen was assayed. Nine infants expired before repeat specimens could be collected, two families declined follow-up, and four screened positive and were referred for confirmatory testing. Of the 60 infants with positive first SCID screenings and adjusted ages of <37 weeks, 41 screened negative for SCID when subsequent NBS specimens were assayed. Sixteen infants expired before repeat specimens could be collected, and three screened positive and were referred for confirmatory testing (Table 4).

Twenty-three of the 46 infants referred for confirmatory testing were found to be false positives. Thirteen were found to have varying types of T-cell lymphopenia (idiopathic, secondary, or transient). Five infants had 22q11.2 deletion syndrome, and two had cartilage–hair hypoplasia. The three remaining infants were diagnosed with CHARGE syndrome, ataxia telangiectasia, and SCID with homozygous *RAG1* c.2974A > G SCID, one with each condition (Table 3). Based on the clinical flow cytometry reference ranges used, this screening method had a positive predictive value of 2% for true SCID (one case found in 46 positive screens) and 48% for T-cell lymphopenias and associated syndromes (22 cases found in 46 positive screens).

## 4. Discussion

Our program successfully replaced a reporting algorithm that utilized TREC quantities with one that relies on the statistical MoM of the TREC Ct in the Wisconsin newborn population. This change has simplified assays by eliminating the need for known TREC copy number standards. Before making this change, we conducted a validation study to assess the population TREC Ct distribution of our newborns. Once this data set was obtained, we determined the median of our population and set a MoM cutoff for positive SCID screens. The clinical validity of the cutoff was further validated with previously confirmed SCID-positive cases.

When using a MoM value to define the positive screening cutoff, it is necessary to monitor the population median, which could potentially change due to assay fluctuations resulting from reagent lot changes. Using the most current population median in MoM calculations is critical for keeping results consistent across time periods and assay shifts. We regularly evaluate the monthly TREC Ct population medians. From 1 September 2018 to 31 March 2021, the mean of 32 monthly TREC Ct population medians was 30.76 cycles, with a standard deviation of 0.146, indicating good stability of the TREC assay. We have not needed to adjust our SCID screening cutoff since we implemented the use of the MoM for reporting TREC assay results.

Since it is a measure of how far an individual test result deviates from the median, the MoM allows normalization of TREC assay data from different laboratories so that individual test results can be compared regardless of the particular method, assay, or reagents used. Screening laboratories can have meaningful SCID screening cutoff comparisons if screening cutoffs are presented with the MoM.

## Figures and Tables

**Table 1:** Demographic information of newborns screened for severe combined immunodeficiency (SCID).

Category		Number	Percentage
**Sex**	Male	80,388	51.1
	Female	76,692	48.8
	Unknown	92	
**Gestational age**	<37 weeks	12,971	8.3
	≥37 weeks	144,168	91.7
	Unknown	33	
**Birth weight**	<2500 g	11,624	7.4
	≥2500 g	145,533	92.6
	Unknown	15	

**Table 2:** Sequences of the primers and probes used for real-time PCR.

Name	Sequence
TREC Forward Primer	5′-CATGCTGACACCTCTGGTT-3′
TREC Reverse Primer	5′-CGGTGAATGAAGAGCAGACA-3′
*RPP30* Forward Primer	5′-AGATTTGGACCTGCGAGCG-3′
*RPP30* Reverse Primer	5′-GAGCGGCTGTCTCCACAAGT-3′
*SMN* Forward Primer	5′-CTTGTGAAACAAAATGCTTTTTAACATCCAT-3′
*SMN* Reverse Primer	5′-GAATGTGAGCACCTTCCTTCTTTTT-3′
TREC Probe	5′-/56-FAM/ACTCCTGTG/ZEN/CACGGTGATGCATAG/3IABkFQ/-3′
*RPP30* Probe	5′-/5HEX/TTCTGACCT/ZEN/GAAGGCTCTGCGCG/3IABkFQ/-3′
*SMN1* Probe	5′-/5ATTO550N/AGG + GTT + T + C + A + GAC/3IAbRQSp/-3′
*SMN2* Blocker	5′-AG + G + GTT + T + T + A + GAC-3′

**Table 3:** Statistics for the cutoff establishment specimens.

	TREC		*RPP30*	
	Ct	MoM	Ct	MoM
*N*	2244	2244	2244	2244
Mean	30.762	1.002	24.280	1.002
Median	30.705	1.000	24.243	1.000
Standard deviation	0.794	0.026	0.643	0.027

**Table 4:** Screening results for Wisconsin newborns with follow-up recommended.

Category	Result of Subsequent Screen or Confirmatory Testing	Number	Percentage
**First screen inconclusive**	Negative screen	105	87.5
	Infant expired	9	7.5
	Family declined follow-up	2	1.7
	Positive screen; confirmatory testing recommended (see below)	4	3.3
**First screen positive, age <37 weeks**	Negative screen	41	68.3
	Infant expired	16	26.7
	Positive screen; confirmatory testing recommended (see below)	3	5.0
**Positive screens referred for confirmatory testing**	T-cell lymphopenia	13 ^a^	28.3
	22q11.2 deletion syndrome	5 ^b^	10.9
	Cartilage–hair hypoplasia	2	4.3
	CHARGE syndrome	1	2.2
	Ataxia telangiectasia	1	2.2
	SCID (homozygous *RAG1*,c.2974A > G)	1	2.2
	False positive	23 ^c^	50.0

^a^ Includes two newborns with inconclusive first screens and one with first screen positive, age <37 weeks. ^b^ Includes one newborn with inconclusive first screen. ^c^ Includes one newborn with inconclusive first screen and two with first screens positive, age <37 weeks.

## Data Availability

The data are not publicly posted and can be requested by contacting the corresponding author.
